# Editorial: Unravelling the Role of Time in Psychological Contract Processes

**DOI:** 10.3389/fpsyg.2018.00813

**Published:** 2018-05-25

**Authors:** Yannick Griep, Tim Vantilborgh, Samantha D. Hansen, Neil Conway

**Affiliations:** ^1^Department of Psychology, University of Calgary, Calgary, AB, Canada; ^2^Stress Research Institute, Stockholm University, Stockholm, Sweden; ^3^Department of Psychology, Vrije Universiteit Brussel, Brussels, Belgium; ^4^Department of Management, University of Toronto–Scarborough and Rotman School of Management, University of Toronto, Toronto, ON, Canada; ^5^Royal Holloway, University of London, Egham, United Kingdom

**Keywords:** psychological contract, time, dynamics, violation feelings, psychological contract breach

The psychological contract (PC) is considered a critical construct in organizational behavior. Upon organizational entry individuals form a PC containing beliefs about the reciprocal obligations between themselves and their employer (Rousseau, 1989, 2001). The PC is self-regulatory and influences how the employee perceives and interprets past, present, and future interactions with the employer. When employees perceive that their employer has failed to fulfill its obligations (i.e., PC breach) they may develop negative affective reactions (e.g., violation feelings; Morrison and Robinson, 1997), negative attitudes, and negative behaviors (for meta-analyses see Zhao et al., 2007; Bal et al., 2008). While the existing PC literature offers a solid theoretical foundation to understand the relationship between perceptions of PC breach, violation feelings, and employee reactions, most PC research has examined relationships in a static, or contemporaneous, manner and has overlooked the temporal context in which PC breach and employee reactions are interrelated and potentially reinforcing to each other over time (for a general critique see Mitchell and James, 2001). The contemporaneous study of PC breach and employee reactions is problematic because it ignores temporal context and the adjustments that employees make to their attitudes and behaviors over time. As such, the current literature fails to acknowledge that the PC is a dynamic construct that is formed, maintained, disrupted, and repaired over time (e.g., Schalk and Roe, 2007; Tomprou et al., 2015; Hansen and Griep, 2016), and that relationships between PC breach and employee reactions that exist at one point in time may not necessarily exist, or vary in strength, at another point in time (see Hansen and Griep, 2016; Griep and Vantilborgh, 2018). This Research Topic is devoted to advancing the PC field by exploring valuable knowledge concerning the role of time in PC processes. In the following, we lay out some critical areas of inquiry in understanding the role of time, and highlight how the innovative set of papers in this Research Topic illustrate exciting future research directions.

## The role of time in PC processes

Theoretically, PCs have always been considered to be dynamic (Rousseau, 1995), yet scholars have only recently started to examine the PC as a dynamic construct by acknowledging time in their research design (for examples see Conway and Briner, 2002; Solinger et al., 2016; Vantilborgh et al., 2016; Griep and Vantilborgh, 2018). Interest in studying the PC as a dynamic phenomenon gained interest thanks to recent theoretical advances, such as the dynamic model of the psychological contract (Schalk and Roe, 2007) and the post-violation model (Tomprou et al., 2015). These novel theoretical insights call for increased attention to studying how the PC is formed, maintained, disrupted, and repaired over time. Following from these theoretical advancements, we identify three critical implications for future research. First, by acknowledging the role of time, it becomes possible for future research to truly capture *processes* or patterns of change in the PC following perceptions of breach. Second, a temporal perspective encourages study of qualitatively distinct PC states and patterns or trajectories of PC reactions that may *emerge* over time (Kozlowski et al., 2013). Third, the inclusion of time in PC research also creates novel *methodological* challenges.

## Capturing processes

A within-person process perspective is critical to understand how perceptions of PC breach and feelings of violation lead to downstream consequences for employees and their organizations. However, the PC literature has relied almost exclusively on a between-person perspective to explore how the attitudes and behaviors of employees who perceived large PC breaches differed from those of employees who perceived no or small breaches (Zhao et al., 2007; Bal et al., 2008). Although useful, such a between-person perspective does not address within-person processes such as how attitudes and behaviors change over time or in reaction to events such as PC breach. Further, longitudinal or experience sampling designs are necessary to examine reciprocal effects, potentially leading to the discovery of unacknowledged relationships (e.g., Griep and Vantilborgh, 2018) and dynamic systems (Vallacher et al., 2002) in the literature. In this Research Topic, both Achnak et al. and Gibbard et al. adopt a within-person process perspective.

Achnak et al. focus on explaining the process leading from breach perceptions to stress experiences, thus contributing to the literature on the effects of PC breach on wellbeing. These consequences for wellbeing have received scarce attention to date, but studies show that PC breach relates positively to emotional exhaustion (Gakovic and Tetrick, 2003; Johnson and O'Leary-Kelly, 2003), anxiety and depression (Conway and Briner, 2002; Slack, 2005), and burnout (Brown, 2007). Across two studies, Achnak et al. show that employees may develop stress over time in response to perceptions of PC breach and that this relationship is mediated by their affective responses. Moreover, Achnak et al. found evidence for the moderating role of fatigue on this positive relationship between perceptions of PC breach and the development of stress, implying that employees who are fatigued at work are at even higher risk to develop stress in response to perceptions of PC breach over time. Interestingly, Achnak et al. also show that over-fulfillment—that is, a positive discrepancy in which an employee receives more than what was obligated—can induce stress.

Gibbard et al. focus on the processes linking team perceptions of PC breach to team performance and effectiveness. They move away from the traditional focus on the individual employee-employer relationship in PC research. They argue that organizations increasingly rely on teams, drawing on the expertise many to generate more innovative solutions than what individual members could have accomplished (Fay et al., 2015; Salas et al., 2015). Within this team context, Marks (2001) has suggested that PCs in work teams may be more impactful than traditional employee-employer PCs because employees are more dependent on their fellow team members to successfully complete a task than they are dependent on their organization as a whole. Although early scholars acknowledged that groups of individuals can develop a shared PC (see Rousseau, 1989, 1995), the idea of shared PCs has historically received little empirical attention. More recently, scholars have devoted substantial attention to the social context as an important factor that influences PC evaluation and the emergence of a team-level PC (e.g., Ho and Levesque, 2005; Dabos and Rousseau, 2013; De Vos and Tekleab, 2014; O'Leary-Kelly et al., 2014; Laulié and Tekleab, 2016). Gibbard et al. s' further understanding of shared perceptions of PC breach by demonstrating that such perceptions are not necessarily detrimental to a team's performance over time, as was once assumed. Gibbard et al. found that shared perceptions of PC breach may generate a context in which team members experience an optimal combination of the desire to be similar to their team while at the same time feeling like they are a unique addition to the team.

## Emergence of PC states and patterns of reactions

Treating the PC as a dynamic phenomenon implies that the PC emerges and changes over time. It also requires researchers to think in terms of patterns or trajectories of reactions to PC breach over time. This focus on emergence is evident when studying how employees cope with perceptions of PC breach and violation (i.e., a severe PC breach) in relation to violation resolution and post-violation PC states. Because most research has solely focused on the negative impact of PC breach and violation feelings on employee attitudes and behaviors (Zhao et al., 2007; Bal et al., 2008), there is limited understanding about how different coping processes may lead to PC restoration and more or less functional post-violation PC states. To fill this void in the literature, Tomprou et al. (2015) developed a conceptual model of post-violation processes in which they detailed how employees may use problem-focused coping strategies to deal with, and attempt to move past, a PC violation over time. In a nutshell, this theory assumes that, through the use of problem-focused coping strategies, employees who experience PC violation are more likely to experience reactivation (i.e., PC that is similar to the pre-violation PC) or thriving (i.e., PC that is more favorable than the pre-violation PC) post-violation PC states, whereas they are less likely to experience impairment (i.e., PC that is less favorable to the pre-violation PC) or dissolution (i.e., mental and behavioral disengagement from the PC) post-violation PC states. Schalk et al.'s study focusses on two types of qualitative data (interviews and archival case studies) to understand different ways in which employees express dissent about PC breach and violation, which strategies they use to cope over time, and what effect these coping strategies have on violation resolution and post-violation PC states. Their findings challenge the unilateral positive view on problem-focused coping as the key to PC restoration by the post-violation model (Tomprou et al., 2015). In contrast, Schalk et al. propose a more nuanced approach to the use of problem-focused coping in relation to PC restoration. Their findings indicated that the use of threatening forms of problem-focused coping (e.g., threatening resignation) was often related to dysfunctional post-violation PC states, whereas the use of competent problem-focused coping (e.g., direct factual appeal), was often, although not always, linked to functional post-violation PC states.

In contrast, Jonas and Griep's conceptual paper introduces a temporal perspective to better understand emerging patterns of reactions to ideological PC breach. Ideological PCs have been called the “third dimension” of the PC (Scheel and Mohr, 2013) and have been defined as the shared mutual agreements between employees and their organization that are built on a set of shared values, mission, and/or purpose that the organization is believed to strive for (Thompson and Bunderson, 2003). These PCs are unique from the typical transactional (i.e., economic or materialistic, tangible, specific, static, and short-term) and relational (i.e., relationship-oriented, intangible, subjective, flexible, and long-lasting) PCs in that negotiation of obligations is focused on a larger shared ideology that is culturally or socially understood rather than a focus on the individual employee-organization interactions (Thompson and Bunderson, 2003). Although a growing number of studies attends to the importance of these ideological PCs as drivers of employee engagement (e.g., Bunderson, 2001; Bunderson and Thompson, 2009; McCabe and Sambrook, 2013; Vantilborgh et al., 2014, 2016), there are questions with regard to why reactions to ideological PC breach appear somewhat inconsistent with mainstream PC theory. For example, research shows that employees react with increased, rather than decreased, work effort in response to perceptions of ideological PC breach (Vantilborgh et al., 2014). Jones and Griep's conceptual model theoretically connects perceptions of ideological PC breach, increases in work effort, and the potential “dark side” of repeated occurrences of ideological PC breach for employees' development of burnout. These authors argue that time plays a central role in the unfolding of employees' reactions to ideological PC breach over time. Specifically, Jones and Griep propose that, as perceptions of ideological PC breach accumulate over time and employees continue to increase their work effort in response, they become more susceptible to burnout.

## Overcoming methodological challenges

Finally, treating the PC as a dynamic phenomenon leads to new methodological challenges that must be overcome. For example, the choice of time lags or the operationalization of constructs can be challenging for researchers setting up experience sampling studies. In the PC literature, the operationalization of PC breach and feelings of violation has been a continual source of debate, which has only become more complex by the introduction of a temporal perspective. In their conceptual model, Morrison and Robinson (1997) clearly distinguished perceptions of PC breach, referring to an employee's perception of unmet employer promises, from violation feelings, referring to the *ensuing* negative emotional state. Violation feelings thus result from a two-stage process in which employees first engage in a cognitive evaluation of a perceived deviation from employer promises, after which negative emotions might follow, which in turn may have several negative attitudinal and behavioral consequences. Despite the general awareness that violation feelings result from said two-stage process, the literature to date has large ignored this process when operationalizing breach and violation (for some exceptions see Solinger et al., 2016; Bal et al., 2017; Griep and Vantilborgh, 2018). This is an important omission because correctly understanding PC processes requires theories, research methods, and statistical models that explicitly recognize that violation feelings follow from an event that exceeds one's acceptance limits, after which a cognitive process determines the intensity of violation feelings. Although dynamic PC theories (Schalk and Roe, 2007; Tomprou et al., 2015) and dynamic research methods (e.g., daily diary, event-sampling, and experience sampling research, Conway and Briner, 2002; Ohly et al., 2010) exist, still lacking are adequate statistical tools to model the above described two-stage process.

Hofmans' study introduces two statistical models—the Zero-Inflated model and the Hurdle model—that closely mimic the theoretical process of perceptions of PC breach and violation feelings via two-stage analytical processes: a binary distribution that models whether PC breach has occurred or not, and a count distribution that models the severity of the negative impact. In doing so, Hofmans contributes to a theoretical-methodological synergy by demonstrating how the application of different methodological techniques can be used to examine an important theoretical issue in PC research. Moreover, this paper will undoubtedly help researchers aiming to measure breach and feelings of violation as dynamic phenomena, by establishing clear guidelines and offering a practical tool for their methods toolbox.

## Conclusion

In conclusion, we believe that it is an exciting time to be doing research on psychological contract processes. The introduction of time in PC processes opens up interesting new avenues for research. The set of papers presented in this Research Topic help provide a better understanding of how PC processes unravel over time (Achnak et al.; Gibbard et al.), how PC states and patterns of reactions emerge over time (Jonas and Griep; Schalk et al.), and how PC breach and feelings of violation can be operationalized and analyzed in novel ways that advance our theoretical understanding of dynamic PC processes (Hofmans). We hope that this collection of papers generates interest among researchers to further incorporate time in the study of PC processes.

## Author contributions

Each author contributed to the writing and conceptualization of this Editorial. The order of contribution is in line with the authorship order.

### Conflict of interest statement

The authors declare that the research was conducted in the absence of any commercial or financial relationships that could be construed as a potential conflict of interest.

## References

[B1] Bal P. M. De Lange A. H. Jansen P. G. W. Van der Velde M. E. (2008). Psychological contract breach and job attitudes: a meta-analysis of age as a moderator. J. Vocat. Behav. 72, 143–158. 10.1080/1359432X.2011.626198

[B2] Bal P. M. Hofmans J. Polat T. (2017). Breaking psychological contracts with the burden of workload: a weekly study of job resources as moderators. Appl. Psychol. 66, 143–167. 10.1111/apps.12079

[B3] Brown L. A. (2007). Extra role time organizational citizenship behavior, expectations for reciprocity, and burnout: potential organizational influence via organizational support and psychological contract fulfillment. Dissert. Abstr. Int. Sect. A Hum. Soc. Sci. 68:793.

[B4] Bunderson J. S. (2001). How work ideologies shape the psychological contracts of professional employees: doctors' responses to perceived breach. J. Organ. Behav. 22, 717–741. 10.1002/job.112

[B5] Bunderson J. S. Thompson J. A. (2009). The call of the wild: zookeepers, callings, and the double-edged sword of deeply meaningful work. Adm. Sci. Q. 54, 32–57. 10.2189/asqu.2009.54.1.32

[B6] Conway N. Briner R. B. (2002). A daily diary study of affective responses to psychological contract breach and exceeded promises. J. Organ. Behav. 23, 287–302. 10.1002/job.139

[B7] Dabos G. E. Rousseau D. M. (2013). Psychological contracts and informal networks in organizations: the effects of social status and local ties. Hum. Resour. Manage. 52, 485–510. 10.1002/hrm.21540

[B8] De Vos A. Tekleab A. G. (2014). Leaders' and employees' psychological contract fulfillment in teams. Acad. Manage. Proc. 2014:12928 10.5465/AMBPP.2014.12928abstract

[B9] Fay D. Shipton H. West M. A. Patterson M. (2015). Teamwork and organizational innovation: the moderating role of the HRM context. Creat. Innov. Manage. 24, 261–277. 10.1111/caim.12100

[B10] Gakovic A. Tetrick L. E. (2003). Psychological contract breach as a source of strain for employees. J. Bus. Psychol. 18, 235–246. 10.1023/A:1027301232116

[B11] Griep Y. Vantilborgh T. (2018). Reciprocal effects of psychological contract breach on counterproductive and organizational citizenship behaviors: the role of time. J. Vocat. Behav. 104, 141–153. 10.1016/j.jvb.2017.10.013

[B12] Hansen S. D. Griep Y. (2016). Psychological contracts, in Handbook of Employee Commitment, ed Meyer J. (Northampton, MA: Edward Elgar Publishers, Inc), 119–135.

[B13] Ho V. T. Levesque L. L. (2005). With a little help from my friends (and substitutes): social referents and influence in psychological contract fulfillment. Organ. Sci. 16, 275–289. 10.1287/orsc.1050.0121

[B14] Johnson J. L. O'Leary-Kelly A. M. (2003). The effects of psychological contract breach and organizational cynicism: not all social exchange violations are created equal. J. Organ. Behav. 24, 627–647. 10.1002/job.207

[B15] Kozlowski S. W. J. Chao G. T. Grand J. A. Braun M. T. Kuljanin G. (2013). Advancing multilevel research design: capturing the dynamics of emergence. Organ. Res. Methods 16, 581–615. 10.1177/1094428113493119

[B16] Laulié L. Tekleab A. G. (2016). A multi-level theory of psychological contract fulfillment in teams. Group Organ. Manage. 41, 658–698, 856 10.1177/1059601116668972

[B17] Marks A. (2001). Developing a multiple foci conceptualization of the psychological contract. Employee Relat. 23, 454–469. 10.1108/EUM0000000005897

[B18] McCabe T. Sambrook S. (2013). Psychological contracts and commitment amongst nurses and nurse managers: a discourse analysis. Int. J. Nurs. Stud. 50, 954–967. 10.1016/j.ijnurstu.2012.11.012 23228863

[B19] Mitchell T. R. James L. R. (2001). Building better theory: time and the specification of when things happen. Acad. Manage. Rev. 26, 530–547. 10.5465/AMR.2001.5393889

[B20] Morrison E. W. Robinson S. L. (1997). When employees feel betrayed: a model of how psychological contract violation develops. Acad. Manage. Rev. 22, 226–256. 10.5465/AMR.1997.9707180265

[B21] O'Leary-Kelly A. M. Henderson K. E. Anand V. Ashforth B. E. (2014). Psychological contracts in a nontraditional industry: exploring the implications for psychological contract development. Group Organ. Manage. 39, 326–360. 10.1177/1059601114525851

[B22] Ohly S. Sonnentag S. Niessen C. Zapf D. (2010). Diary studies in organizational research. J. Pers. Psychol. 9, 79–93. 10.1027/1866-5888/a000009

[B23] Rousseau D. (1995). Psychological Contracts in Organizations: Understanding Written and Unwritten Agreements. Thousand Oaks, CA: Sage Publications.

[B24] Rousseau D. M. (1989). Psychological and implied contracts in organizations. Employee Responsibil. Rights J. 2, 121–139. 10.1007/BF01384942

[B25] Rousseau D. M. (2001). Schema, promise and mutuality: the building blocks of the psychological contract. J. Occup. Organ. Psychol. 74, 511–541. 10.1348/096317901167505

[B26] Salas E. Shuffler M. L. Thayer A. L. Bedwell W. L. Lazzara E. H. (2015). Understanding and improving teamwork in organizations: a scientifically based practical guide. Hum. Resour. Manage. 54, 599–622. 10.1002/hrm.21628

[B27] Schalk R. Roe R. E. (2007). Towards a dynamic model of the psychological contract. J. Theory Soc. Behav. 37, 167–182. 10.1111/j.1468-5914.2007.00330.x

[B28] Scheel T. Mohr G. (2013). The third dimension: value-oriented contents in psychological contracts. Eur. J. Work Organ. Psychol. 22, 390–407. 10.1080/1359432X.2012.665229

[B29] Slack K. J. (2005). Examining job insecurity and well-being in the context of the role of employment. Dissert. Abstr. Int. Sect. B Sci. Eng. 65:4884.

[B30] Solinger O. N. Hofmans J. Bal P. M. Jansen P. G. W. (2016). Bouncing back from psychological contract breach: how commitment recovers over time. J. Organ. Behav. 37, 494–514. 10.1002/job.2047

[B31] Thompson J. A. Bunderson J. S. (2003). Violations of principle: ideological currency in the psychological contract. Acad. Manage. Rev. 28, 571–586. 10.5465/AMR.2003.10899381

[B32] Tomprou M. Hansen D. S. Rousseau M. D. (2015). The psychological contracts of violation victims: a post-violation model. J. Organ. Behav. 36, 561–581. 10.1002/job.1997

[B33] Vallacher R. Read S. Nowak A. (2002). The dynamical perspective in personality and social psychology. Pers. Soc. Psychol. Rev. 6, 264–273. 10.1207/S15327957PSPR0604

[B34] Vantilborgh T. Bidee J. Pepermans R. Griep Y. Hofmans J. (2016). Antecedents of psychological contract breach: the role of job demands, job resources, and affect. PLoS ONE 11:e0154696. 10.1371/journal.pone.0154696 27171275 PMC4865204

[B35] Vantilborgh T. Bidee J. Pepermans R. Willems J. Huybrechts G. Jegers M. (2014). Effects of ideological and relational psychological contract breach and fulfilment on volunteers' work effort. Eur. J. Work Organ. Psychol. 23, 217–230. 10.1080/1359432X.2012.740170

[B36] Zhao H. Wayne S. J. Glibkowski B. C. Bravo J. (2007). The impact of psychological contract breach on work-related outcomes: a meta-analysis. Pers. Psychol. 60, 647–680. 10.1111/j.1744-6570.2007.00087.x

